# Antimony resistance in *Leishmania* (*Viannia*) *braziliensis* clinical isolates from atypical lesions associates with increased ARM56/ARM58 transcripts and reduced drug uptake

**DOI:** 10.1590/0074-02760190111

**Published:** 2019-08-15

**Authors:** Jeronimo Nunes Rugani, Célia Maria Ferreira Gontijo, Frédéric Frézard, Rodrigo Pedro Soares, Rubens Lima do Monte-Neto

**Affiliations:** 1 Fundação Oswaldo Cruz-Fiocruz, Instituto René Rachou, Belo Horizonte, MG, Brasil; 2 Universidade Federal de Minas Gerais, Instituto de Ciências Biológicas, Departamento de Fisiologia e Biofísica, Belo Horizonte, MG, Brasil

**Keywords:** antimony resistance, Leishmania braziliensis, field isolates, antimony resistance marker, ARM56, ARM58, atypical lesions

## Abstract

**BACKGROUND:**

In addition to the limited therapeutic arsenal and the side effects of antileishmanial agents, drug resistance hinders disease control. In Brazil, *Leishmania braziliensis* causes atypical (AT) tegumentary leishmaniasis lesions, frequently refractory to treatment.

**OBJECTIVES:**

The main goal of this study was to characterise antimony (Sb)-resistant (SbR) *L. braziliensis* strains obtained from patients living in Xakriabá indigenous community, Minas Gerais, Brazil.

**METHODS:**

The aquaglyceroporin 1-encoding gene (AQP1) from *L. braziliensis* clinical isolates was sequenced, and its function was evaluated by hypo-osmotic shock. mRNA levels of genes associated with Sb resistance were measured by quantitative reverse transcription polymerase chain reaction (qRT-PCR). Atomic absorption was used to measure Sb uptake.

**FINDINGS:**

Although clinical isolates presented delayed recovery time in hypo-osmotic shock, AQP1 function was maintained. Isolate 340 accumulated less Sb than all other isolates, supporting the 65-fold downregulation of AQP1 mRNA levels. Both 330 and 340 isolates upregulated antimony resistance marker (ARM) 56/ARM58 and multidrug resistant protein A (MRPA); however, only ARM58 upregulation was an exclusive feature of SbR field isolates. CA7AE seemed to increase drug uptake in *L. braziliensis* and represented a tool to study the role of glycoconjugates in Sb transport.

**MAIN CONCLUSIONS:**

There is a clear correlation between ARM56/58 upregulation and Sb resistance in AT-harbouring patients, suggesting the use of these markers as potential indicators to help the treatment choice and outcome, preventing therapeutic failure.

*Leishmania (Viannia) braziliensis* causes cutaneous leishmaniasis (CL), severe mucocutaneous leishmaniasis (MCL), and unusual cutaneous manifestations. Unlike typical CL lesions ― commonly classified by a well-defined and limited oval ulcer, atypical (AT) tegumentary leishmaniasis, is recognised by dry, verrucous, and sporotrichoid lesions, which leads to misdiagnosis due to similarities with other dermatological pathologies.^1^ ^,^ ^2^ ^,^ ^3^ In Brazil, AT lesions were reported in Minas Gerais and Bahia states.^1^ ^,^ ^3^ ^,^ ^4^ The histopathology of AT lesions caused by *L. braziliensis*, similar to classical CL lesions, shows infiltrated plasma cells and lymphocytes with few or none parasites.^1^ AT and CL lesions present similar inflammatory cytokine levels, differing only if the lesion has been formed more recently or later. In more recent lesions, there is high expression of a panel of pro-inflammatory cytokines, while modulatory cytokines are characteristic of later lesions.^2^ ^,^ ^5^ In addition, *L. braziliensis* AT-derived clinical isolates are associated with a variant genetic profile, showing a clustered distribution in population structure analysis.^4^

We have recently observed that AT lesion-derived *L. braziliensis* strains are highly resistant to antimony (Sb), supporting the treatment failure observed in *L. braziliensis* Sb-resistant (SbR) infected patients who received glucantime as chemotherapy.^3^ Sb resistance was first described in India for *Leishmania donovani*-causing visceral leishmaniasis (VL),^6^ and treatment refractory CL due to species from the *L. (Viannia)* subgenus, including *L. braziliensis*, were reported in South America.^2^ ^,^ ^7^ *L. braziliensis* is commonly more sensitive to Sb than other species. However, *L. braziliensis* isolated from patients with treatment failure history derived from both CL typical or AT lesions were found to be less sensitive to treatment compared to isolates from patients with clinical cure.^3^

To avoid the spread of drug resistance, Sb resistant strains should be identified before treatment is chosen and administered. Therefore, the molecular mechanisms associated with the resistance phenotype in field isolates should be characterised so that select key markers can be screened for drug resistance in the field. In *Leishmania* parasites, Sb resistance is due to decreased drug uptake, increased drug efflux, or drug neutralisation strategies that pump active drug in intracellular vesicles or inactivate the drug through thiol binding followed by Sb extrusion.^8^ *Leishmania* parasites can become resistant to Sb by reducing expression or mutating the aquaglyceroporin 1-encoding gene (AQP1). This porin is the main entry route of Sb into the cell, and its disruption ― achieved by gene deletion or point mutation ― culminates with non-functional AQP1 unable to mediate Sb uptake and intracellular Sb accumulation. A similar effect can also be observed upon downregulation of AQP1 leading to diminished Sb levels within the parasite.^9^ ^,^ ^10^ ^,^ ^11^ The same phenotype is reached when Sb is a substrate of the ABC transporters and can be pumped out or transported into intracellular vesicles, followed by extrusion. Several transporter proteins were associated with Sb resistance in different *Leishmania* species, from both field isolates and laboratory-selected resistant mutants such as, multidrug resistant protein A (MRPA)/ABCC3 (also known as PGPA),^12^ ^,^ ^13^ ABCG2,^14^ ABCI4,^15^ and antimony resistance marker (ARM)56/ARM58.^16^ However, the roles of such resistance genes are still unknown for *L. braziliensis* isolates derived from AT lesions.

Here, we explored molecular markers associated with Sb resistance in *L. braziliensis* clinical isolates from an Indigenous community in the north of Minas Gerais state, Brazil. Sb-resistant strains were obtained from AT-harbouring patients refractory to glucantime treatment. Functional analysis, involving drug transport and osmoregulation, was performed in order to validate and characterise previously identified Sb resistance.

## MATERIALS AND METHODS

*Parasites* - Promastigote forms of *L.* (*Viannia*) *braziliensis* isolated from classical CL lesion (strains MHOM/BR/2008/**426** and MHOM/BR/2009/**346**), derived from AT lesions (strains MHOM/BR/2008/**330** and MHOM/BR/2008/**340**), reference strain MHOM/BR/1975/**M2903,** and *L.* (*Leishmania*) *infantum* Δ*lpg* (strain MCAN/BR/1989/**BA262**)^17^ were maintained in a-MEM medium (Gibco/Thermo Fisher) supplemented with 10% foetal bovine serum, 5 μg/mL of hemin, and 5 μM of biopterin in pH 7 at 26ºC. Field isolates were previously typed as *L. braziliensis*.^3^ All clinical isolates were obtained prior to patient treatment, meaning that isolates from AT lesions were intrinsically resistant to Sb.^4^ The resistance was not acquired during Sb exposition in these patients. Patients were submitted to one round of treatment with 20 mg Sb^V^ (glucantime as source of Sb^V^)/kg/day for 20 days. The patients who presented AT lesions were refractory and submitted to a second round of treatment to achieve cure. One of the patients ― from whom the strain 340 was isolated ― dropped out the therapeutic scheme.^3^ The resistance index of AT lesion-derived *L. braziliensis* was > 12-fold when compared to reference strain M2903.^3^

*Hypo-osmotic shock assay* - Hypo-osmotic stress was applied as a way to assess cell volume measurements and infer about osmoregulation, as previously described.^18^ Briefly, mid-log phase *L. braziliensis* promastigotes were washed twice in HEPES/NaCl/Glucose buffer (20 mM HEPES, 0.15 M NaCl, 10 mM glucose, pH 7.2), adjusted to a final volume of 100 mL containing 1 × 10^8^ parasites/mL, and incubated in a 96-well flat bottom plate. Hypo-osmotic shock was induced by adding equal volume of Milli-Q water to the isotonic parasite culture, followed by absorbance measurement at 550 nm during 5 min with 9 s interval between reads in a microplate reader (Spectramax M5, Molecular Devices). The culture dilution leads to changes in osmolarity (cellular hypo-osmotic stress) leading the cellular volume increase that corresponds to a reduction in absorbance. Isosmotic control was established by adding equal volume of saline buffer to the cultures. Absorbance measures were plotted using GraphPad Prism version 6.0 as normalised values by subtracting first read value from all time point reads. The experiments were performed three times in triplicate.

*AQP1 sequencing* - Genomic DNA of mid-log growth phase *L. braziliensis* promastigotes was extracted using Gentra Puregene Cell and Tissue Kit (QIAGEN #158667) according to manufacturer’s instructions. Genomic DNA was used to amplify AQP1 (*LbrM.31.0020*) by PCR using the following forward and reverse primers: 5’-ATGGCGATTGAAAACCACATGG-3’ and 5’-CTACGCACCGCTCGGTATTATA-3’, resulting in an amplicon that comprises the whole ORF of 858 bp. The resulted amplicon was purified using QIAquick polymerase chain reaction (PCR) purification kit (QIAGEN #28104), cloned into pGEM-T easy vector (Promega, Madison, WI, USA), and Sanger sequenced in a capillary sequencer ABI3730xl DNA Analyzer (ThermoFischer) using SP6 (ATTTAGGTGACACTATAG) and T7 (TAATACGACTCACTATAGGG) primers. All sequences were analysed using FinchTV software (Geospiza, INC.), and amplicon sequences were aligned with sequences obtained from GenBank using the BLAST tool.^8^ The consensus sequences were aligned with *L. braziliensis* M2904 AQP1 gene (*LbrM.31.0020*) using MEGA X software to analyse the presence of single nucleotide polymorphisms (SNPs) and possible amino acid substitutions.

*Intracellular antimony measurement* - The Sb uptake assay was performed as previously reported.^19^ Mid-log growth phase *Leishmania* promastigotes were washed with HEPES/NaCl/Glucose buffer, and adjusted to a population of 1 × 10^8^ cells/mL. Parasites (1 × 10^8^) were incubated with 500 μM of Sb^III^ (potassium antimonyl tartrate, Sigma Aldrich #244791) for 1 h at 26ºC in a 24-well plate. The cultures were transferred to microtubes, placed in ice for Sb transport blocking, and washed four times with 1 mL of HEPES/NaCl/Glucose ice-cold buffer at 1816 *g* for 5 min at 4ºC. The resulting pellet was digested overnight with 100 μL of 65% HNO_3_ (Merck Millipore #100456). Intracellular Sb trace content was quantified by graphite furnace electrothermal atomic absorption spectrometry using an AAnalyst 600/800 spectrometer (Perkin Elmer, Waltham, MA, USA). Blank matrix was established as promastigotes without drug exposition, while the adsorption control was exposed to Sb at 4ºC and washed immediately after drug exposure. Blank measure values were subtracted as background and intracellular Sb content was normalised by the number of cells. To perform the uptake kinetics, we incubated the parasites with the CA7AE antibody (1:400), which recognises the Gal(β1,4)Man(α1)-PO_4_ repeat units present in units present in lipophosphoglycan (LPG) surface molecule. All assays were performed three times as independent experiments with four replicates each.

*Real-time reverse transcription PCR (RT-PCR)* - Total RNA was extracted from mid-log phase promastigotes, using the RNeasy Plus Mini Kit (Qiagen #74134), where 5 μg was used to synthesise cDNA following manufacturer’s instructions of Super-Script First Strand Synthesis System for RT-PCR (ThermoFischer #11904018). This cDNA was used as a template in reactions containing 1× iTaq Universal SYBR Green Supermix (Bio-Rad #10000068167), 100 nM forward and reverse primers, and 100 ng of cDNA target. The reaction was carried out in a program of 95ºC for 5 min, and then cycled 40 times at 95ºC for 15 s and 60ºC for 30 s. The assays were performed in three biological replicates. Relative amounts of PCR products generated from each primer set were determined based on the cycle threshold (Ct) value and amplification efficiencies. Data were analysed using the comparative 2^-ΔΔCt^ method, where GAPDH (glyceraldehyde-3-phosphate dehydrogenase) coding gene (*LbrM.30.2950*) was used as a housekeeping gene. All primers for targeted genes and their sequences are listed in Table I.

**TABLE I:** Primers used in quantitative real-time polymerase chain reaction (PCR)

Target gene (ID)	Sequence 5’- 3’ (Forward and reverse)	Tm	GC %	Amplicon length (bp)
GAPDH (*LbrM.30.2950*)	CGACCAGGACCTTATTGGTAAA	63.7	45.5	100
	CACCGTATCGTGCTTCATCT	62.7	50	
MRPA (*LbrM.23.0280*)	CTTCTCCATGTGCTCCACTATC	62.6	50	103
	CACCTGCATCAGCTTGTAGTA	60.5	47.6	
AQP1 (*LbrM.31.0020*)	GGTATCACGACAGGTATCAACTC	61.3	47.8	84
	CCAGAGCATGGCTGTGAATA	63.8	50	
GSH1 (*LbrM.18.1700*)	GGGTGGCTTCTATGGACTTATAC	61.5	47.8	100
	CATGGACAGGAACCTCAAGTAG	62.5	50	
ABCG2 (*LbrM.06.0080*)	CTGAGTTTCCCGTGCAGATT	64.2	50	107
	CACACCGCAGTAGTAGAAGAAC	60.4	50	
ABCI4 (*LbrM.33.3540*)	CTGTAGACGAAGCGGGTATTT	62	47.6	135
	CTAGGCGATGAGACACCATAAC	62.4	50	
AMR56 (*LbrM.20.0200*)	GAGAACGGACAACCCAGTTT	62.9	50	105
	CCACTTCTTCTGCTGTGAGTT	61.1	47.6	
ARM58 (*LbrM.20.0210*)	CCCAAGGGCTTTCACCTAAA	64.9	50	103
	AGCGGTAGATCTTGTCGTATTG	62	45.5	

GAPDH: glycosomal glyceraldehyde 3-phosphate dehydrogenase; MRPA: multidrug resistant protein A (ABC transporter ABCC3, also known as PGPA); AQP1: aquaglyceroporin 1; GSH1: gamma-glutamylcysteine synthetase; ABCG2: ATP-binding cassette protein subfamily G, member 2; ABCI4: ATP-binding cassette protein subfamily I member 4; ARM56 and ARM58: antimony resistance marker of 56 and 58 kDa. bp: base pair. Tm (melting temperature) was calculated at default settings of 0.25 mM oligo concentration and 50 mM Na. Primers were designed using PrimerQuest (www.idtdna.com/site/account/login?returnurl=%2FPrimerquest%2FHome%2FIndex), rechecked with OligoAnalyzer (https://www.idtdna.com/calc/analyzer) and OligoEvaluator (Guimarães et al.,^(1,2)^ Rugani et al.^(3)^).

*Ethics* - The *L. braziliensis* clinical isolates were obtained from patients from an indigenous reserve as previously published.^4^ This main project was approved by CONEP nº 355/2008 and FUNAI authorisation nº149/CGEP/08 to access indigenous territory.

## RESULTS

**Fig. 1:**
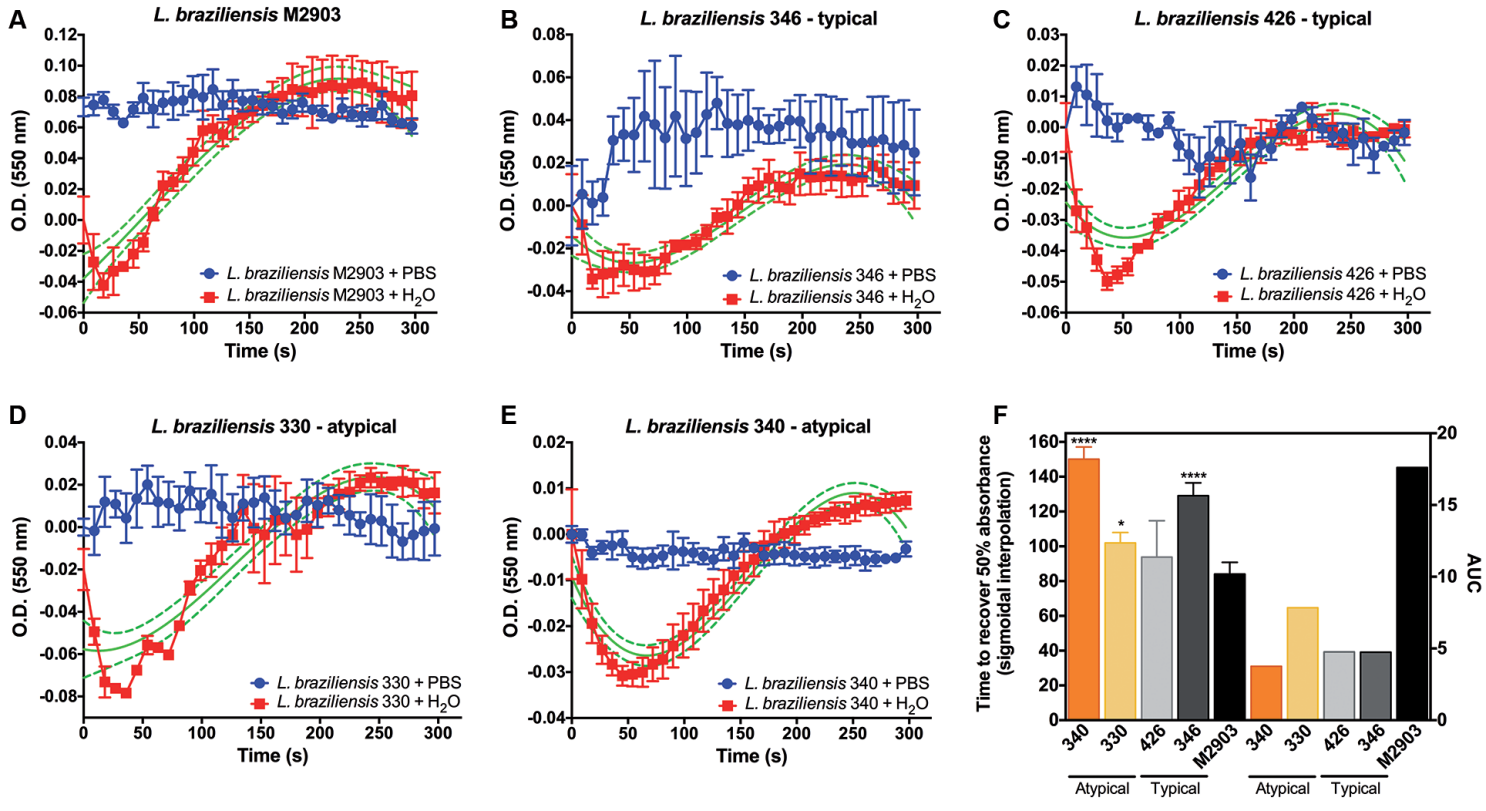
volume regulation of *Leishmania braziliensis* field isolates. Hypo-osmotic shock was performed upon equal volume addition of Milli-Q-water to the cultures (red). Isotonic controls were established by adding isotonic saline buffer (blue). Absorbance was measured at 550 nm during 5 min after hypo-osmotic stress. Increases in cellular volume are represented by reduced absorbance, which is reverted by normal cellular osmoregulation. The recovery time was calculated using non-linear regression functions (sigmoidal, bell shaped, and third order polynomial). Sigmoidal interpolation is represented in green. The time to recover 50% in absorbance was compared using a one-way analysis of variance (ANOVA) followed by Bonferroni’s multiple comparisons test, where ****p < 0.00001 and *p < 0.01, when compared with the reference strain M2903 (F). AUC: area under the curve. *L. braziliensis* strains: M2903 (A); 346 (B); 426 (C); 330 (D); 340 (E). Data are shown as mean ± SE (standard error of the mean) from three independent experiments performed in triplicates.

*L. braziliensis SbR field isolates are able to recover cellular volume upon hypo-osmotic stress but slower than reference strain* - The hypo-osmotic shock assay is a suitable method to evaluate the integrity of aquaglyceroporin function on osmotic control; therefore, we applied this strategy to associate AQP1 defects with the Sb resistance mechanism in *L. braziliensis* field isolates. All strains were able to recover cellular volume upon hypo-osmotic stress (Fig. 1A-E). However, field isolates 346, 330, and 340 presented a delayed recovery time when compared with the M2903 reference strain (Fig. 1F). Based on hypo-osmotic shock assay, we were not able to differentiate among typical or AT lesion-derived strains. However, we clearly see at least a 3-fold reduction in the area under curve from field isolates when compared with M2903 (Fig. 1F), which is directly proportional to the slope and explains the osmoregulation delay.

*SNPs in AQP1 gene coding sequence led to silent mutations and did not associate with SbR in L. braziliensis field isolates* - Once we determined that the AQP1 main function was not disrupted, we decided to investigate its gene coding sequencing integrity, mRNA, and Sb uptake levels as an attempt to implicate or exclude this marker in SbR *L. braziliensis* field isolates. When comparing the AQP1 gene sequence of *L. braziliensis* field isolates with the M2903 and M2904 reference strains, we observed that AT lesion-derived SbR strains, 330 and 340, presented the SNP, A516C; however, this SNP did not alter the protein sequence and was considered a silent mutation [Table II, Supplementary data (Figure)]. The Guanine to Adenine change in the AQP1 gene at position 447 led to a V150A point mutation in the amino acid sequence from typical lesion-derived parasites (Table II). This feature was also shared with M2903, but differed in the M2904 reference strain. Three other SNPs, A372G, T394C, and T449C, were observed in M2903 and all field isolated strains [Table II, Supplementary data (Figure)].

**TABLE II:** Single nucleotide polymorphisms (SNPs) of the AQP1 gene of *Leishmania braziliensis* field isolates and M2903 reference strain

Strain	Lesion	DNA	Aminoacid
M2903	Typical	A372G	S
		T394C	S
		G447A	S
		T449C	V150A
346	Typical	A372G	S
		T394C	S
		G447A	S
		T449C	V150A
426	Typical	A372G	S
		T394C	S
		G447A	S
		T449C	V150A
330	Atypical	A372G	S
		T394C	S
		T449C	S
		A516C	S
340	Atypical	A372G	S
		T394C	S
		T449C	S
		A516C	S

Red: SNP exclusive from AT lesion-derived *L. braziliensis*; blue: SNP present only in typical lesion-derived parasites and shared with M2903, but differs from reference genome of M2904; green: SNPs present in AQP1 from field isolates, shared with M2903, but absent in M2904. S: silent mutation. Primers AQP1F and AQP1R were used for amplification of fragment, and SP6 and T7 for sequencing.

**Fig. 2:**
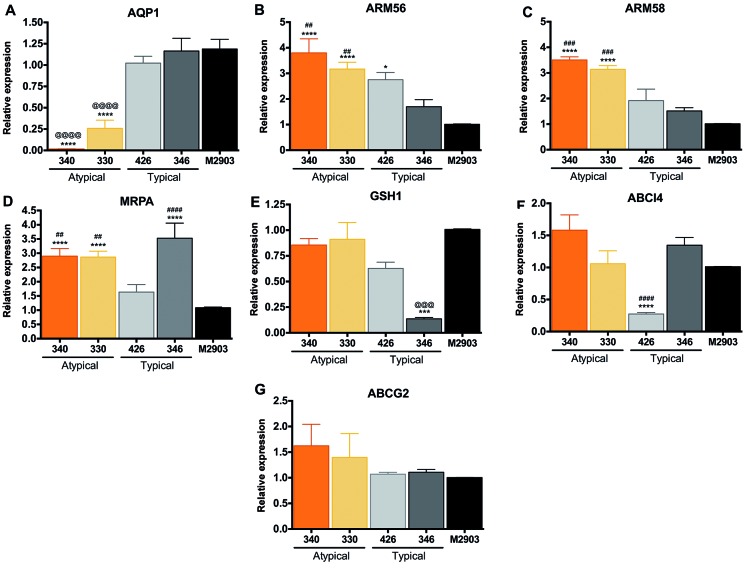
transcript levels of antimony (Sb) resistance-associated genes in *Leishmania braziliensis* field isolates. (A) AQP1: aquaglyceroporin 1. (B) and (C) ARM56 and ARM58: antimony resistance marker of 56 and 58 kDa. (D) MRPA: multidrug resistant protein A (ABC transporter ABCC3, also known as PGPA). (E) GSH1: gamma-glutamylcysteine synthetase. (F) ABCI4: ATP-binding cassette protein subfamily I member 4. (G) ABCG2: ATP-binding cassette protein subfamily G, member 2. Constitutive GAPDH (*LbrM.30.2950*) was applied as housekeeping gene. Values represent the mean of at least two independent measurements performed with three biological replicates. Statistical analysis was performed using one-way analysis of variance (ANOVA) followed by Tukey’s multicomparisons test. Asterisks represents the comparison with the M2903 reference strain, while # or @ symbols indicates the differences between at least one of the SbR or Sb-sensitive strains. *p < 0.01; ***p < 0.0002; ****p < 0.0001.

*SbR L. braziliensis field isolates upregulated ARM56/ARM58 while downregulating AQP1* - Several molecular markers have been associated with Sb resistance in *Leishmania* parasites. Because drug resistance is a multifactorial phenomenon, we decided to investigate the transcript level of not only AQP1, but also Sb resistance-associated targets, such as ARM56 and 58, MRPA, ABCI4, ABCG2, and GSH1. Downregulation in AQP1 was observed in SbR *L. braziliensis* isolates 330 and 340 (Fig. 2A), revealing that reduced Sb uptake could be associated with resistance mechanisms, even if its sequence is preserved. AQP1 was highly downregulated in isolate 340, reaching 65-fold when compared with M2903, while AQP1 mRNA levels of 330 were reduced 3-fold (Fig. 2A). Another clear difference in Sb resistance-associated genes is the upregulation of Sb resistance markers, ARM56/ARM58, respectively, in SbR AT lesion-derived *L. braziliensis* (Fig. 2B-C). ARM58 was exclusively upregulated in SbR parasites, reinforcing its chance of being directly involved in Sb resistance in these isolates (Fig. 2C). Upregulation of drug efflux pump, like ABCC3 (MRPA), was also observed in SbR parasites; however, it was not an exclusive feature of them, discouraging its association with resistance mechanism, because Sb-sensitive 346 upregulated this marker as well (Fig. 2D). Curiously, GSH1 encoding gene mRNA was downregulated in isolate 346 (Fig. 2E). This downregulation could explain Sb sensitivity, because thiol-mediated drug efflux or neutralisation was potentially incipient or absent due to reduced glutathione levels. Downregulation of ABCI4 would also support Sb susceptibility in *L. braziliensis* isolate 426 (Fig. 2F). Among the tested SbR markers, ABCG2 was the only one to remain non-modulated and non-associated with either the sensitivity or resistance mechanisms (Fig. 2G).

**Fig. 3:**
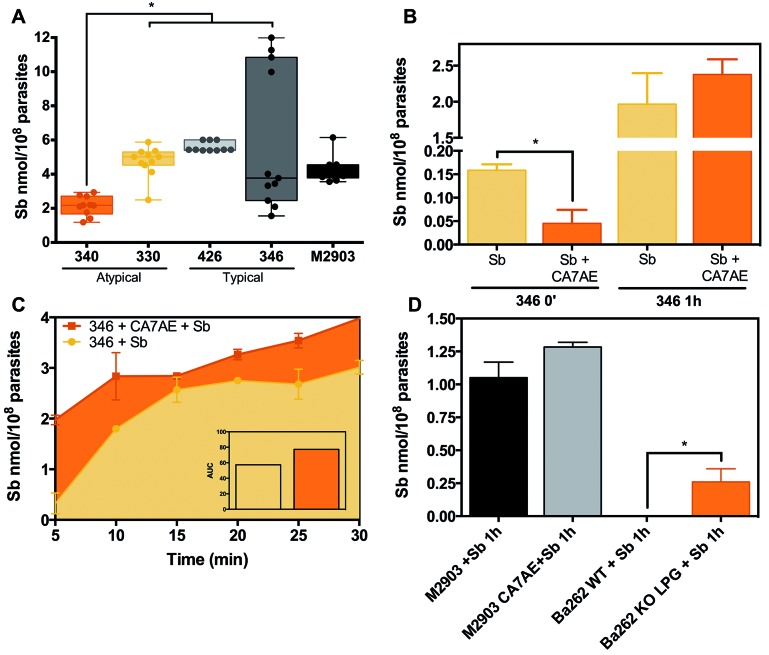
antimony (Sb) uptake in *Leishmania braziliensis* field isolates. Mid-log phase promastigote forms were exposed to 500 mM of Sb^III^ and incubated for 1 h at 26ºC in HEPES/NaCl/Glucose buffer. Cells were washed four times and digested in 65% nitric acid. Intracellular Sb traces were measured by graphite furnace atomic absorption spectrometry. Background matrix was established using only parasites without drug exposition. The average intracellular Sb value was subtracted from adsorption control ― where Sb was added to the culture at 4ºC and immediately washed with ice cold buffer ― and normalised by 1 x 10^8^ parasites. Sb uptake from both AT or typical lesion-derived *L. braziliensis* strains, 330, 340, 346, and 426, was compared with reference strain M2903 (A). Sb uptake in *L. braziliensis* isolate 346 was assayed in the presence of CA7AE antibody, which recognises the Gal(β1,4)Man(α1)-PO_4_ repeat units present in LPG, upon 1 h exposition (B) or in a kinetic curve of early time points from 5 to 30 min (C). Ba262 KO LPG is a *L. infantum* strain where the mannosyltranferase encoding gene was disrupted, and they are not able to assemble LPG molecule (D). This strain was used as a control to infer about LPG interference in Sb uptake. *p < 0.01. Data are shown as mean ± SE (standard error of the mean) obtained from three independent experiments performed in quadruplicates.

*SbR L. braziliensis isolate 340 presented reduced Sb uptake* - Because AQP1 mRNA was reduced in SbR AT lesion-derived *L. braziliensis*, we decided to measure and compare Sb uptake in all field isolates in order to functionally characterise Sb entry transport. Sb incorporation into *L. braziliensis* SbR isolate 340 was reduced when compared to Sb-sensitive strains and even to its counterpart, isolate 330 (Fig. 3A), corroborating what was observed in mRNA expression results and functionally validating this mechanistical observation. Sb-sensitive isolate 346 presented a wide distribution in Sb uptake values among three independent experiments, reaching the highest intracellular Sb levels (Fig. 3A).

*L. braziliensis surface glycoconjugates could interfere with Sb uptake* - The heterogeneity and high levels of intracellular Sb content observed in Sb-sensitive *L. braziliensis* isolate 346 led us to further evaluate differential Sb accumulation for a better understanding of Sb uptake in Sb-sensitive isolates and potentially speculate about Sb transport-dependent drug resistance. For this analysis, we blocked the major *Leishmania* glycoconjugate LPG with CA7AE prior to Sb exposure. After 1 h, we did not see any difference in Sb uptake (Fig. 3A-B), although adsorbed controls varied. Then, we decided to pursue an early time point kinetics (5-30 min). CA7AE-mediated agglutination favours Sb accumulation in isolate 346 (Fig. 3C), at least during early events of its exposure. To validate this observation, we performed similar experiments using a GPI-14 (manosyltransferase) *Leishmania* knockout strain (Ba262 Δ*lpg*). GPI-14 is an essential enzyme in LPG assembling, and this knockout strain resulted in LPG absence in these mutants. As expected, increased Sb uptake was detected in LPG-deficient *Leishmania* Ba262 Δ*lpg* when compared with Ba262 wild-type after a 1 h Sb incubation (Fig. 3D). A similar Sb accumulation pattern was observed for M2903 and 346 upon CA7AE Ab/Sb exposition (Fig. 3D).

## DISCUSSION

Drug resistance is a multifactorial phenomenon, and efforts have been made to unravel common molecular markers of SbR strains. However, the nature of drug resistance together with biochemical heterogeneity among *Leishmania* species complicates this challenge. Because of that, the typing of Sb resistance based on multiple molecular markers identified in field isolates is relevant as a surveillance strategies to control leishmaniasis and to avoid Sb resistance spreading. As a wider study on the assessment of Sb resistance in *Leishmania*, this work focused on AT lesion-derived *L. braziliensis*, in which the Sb resistance index was at least 12-fold when compared to the *L. braziliensis* reference strain.^3^

Because Sb resistance in *Leishmania* parasites usually involves drug transport modulation, in this study, we focused on the molecular and functional characterisation associated with Sb dynamics to mechanistically infer about this phenomenon in *L. braziliensis* clinical isolates. To achieve this goal, we started with the simplest and most cost effective functional way to indirectly evaluate AQP1 function; we added water to the *Leishmania* cell culture, which increased the cell volume, to monitor the osmoregulation-mediated recovery by absorbance. This technique has been used to infer about AQP1 function in trypanosomatids,^18^ and we found that cell recovery volume patterns upon hypo-osmotic shock were useful to distinguish between field and laboratory reference strain, M2903. This difference could be associated with adaptations under axenic culture conditions, because the *L. braziliensis* M2903 reference strain is very well adapted to *in vitro* maintenance compared to fresh (or fewer passages) clinical isolates. Once we determined that AQP1 was the main Sb entry route in *Leishmania* parasites^9^ and was also involved in osmoregulation, we decided to characterise the latter to determine AQP1 integrity and extrapolate to decreased uptake-related Sb resistance. However, despite the differences in recovery time, all parasites were able to re-establish cell volume ― indirect evidence that AQP1 osmoregulation was not compromised. Nonetheless, the solute transport function can be impaired by specific point mutations and be associated with Sb resistance.^10^ Thus, we perform AQP1 gene sequencing, but we were unable to associate any SNPs to Sb resistance, because they were silent mutations, suggesting other mechanisms. However, exclusive SNPs on AQP1 from SbR or Sb-sensitive *L. braziliensis* (A516C and G447A) can be applied to the development of molecular diagnostic strategies that will be useful for drug resistance predictions.

Despite playing a role in osmoregulation, a reduction in AQP1 gene expression is associated with lower Sb^III^ uptake, decreasing the drug concentration into *Leishmania* cells and resulting in a SbR phenotype.^10^ Because we were unable to associate Sb resistance with AQP1 mutations or compromised AQP1 osmoregulation-related function, we decided to verify its mRNA levels. Indeed, only SbR *L. braziliensis* field isolates 330 and 340 presented reduced AQP1 mRNA levels, which were functionally validated by reduced Sb uptake. AQP1 downregulation was also observed in other *Leishmania* species, such as *L. tropica* ^20^ and *L. donovani*,^21^ derived from patients unresponsive to Sb-based treatment. Our data corroborate the reduced expression found not only in field isolates, but also in laboratory-SbR selected mutants.^10^ ^,^ ^19^ There are two studies on *L. braziliensis* isolated from Brazilian patients with different antimonial treatment outcomes showing either unchanged AQP1 levels^22^ or an increase in AQP1 expression.^23^ Eslami et al. also reported increased AQP1 levels in *L. major* isolated from patients refractory to glucantime in Iran.^24^ However, when citing previous work conducted by Dr. Rita Mukhopadhyay’s team,^9^ Eslami et al.^24^ mistakenly affirmed that overexpression of *Lm*AQP1 induces Sb resistance, which is not true and does not correspond to the findings by Gourbal et al.,^9^ which observed a 10-fold Sb^III^ resistance up to one allele *Lm*AQP1 gene disruption. This raises doubts about how the authors in the work of Eslami et al.^24^ interpreted their data. Due to this mosaicism, it is important to establish species-specific markers once resistance is multifactorial. We clearly see an association between AQP1 downregulation in AT lesion-derived *L. braziliensis* isolates and reduced Sb uptake.

Our next step was to compare mRNA levels of genes previously associated with Sb resistance. Because Sb resistance is a dynamic interplay between reduced drug uptake and increased efflux, we selected key transporters implicated in drug extrusion. The ABC transporter, MRPA (ABCC3), formerly known as P-glycoprotein A (PGPA), is responsible for the sequestration of metal-thiol conjugates to an intracellular organelle located near the flagellar pocket.^12^ MRPA is one of the most studied targets in SbR *Leishmania* and is found in both clinical isolates^12^ and laboratory selected mutants.^10^ In our study, MRPA mRNA levels were found to be upregulated in SbR and in typical lesion-derived Sb-sensitive *L. braziliensis* isolate 346. This suggests that MRPA is not directly linked to Sb resistance in *L. braziliensis* obtained from Xakribá indigenous community in Minas Gerais. ABC proteins, including ABCC3/MRPA, have thiols/Sb conjugates as substrates, promoting drug efflux.^8^ ^,^ ^12^ Intracellular Sb can also mediate the reduced thiol buffering capacity, inducing higher reduction environments by feedback towards drug resistance.^8^ MRPA was increased in *L. braziliensis* isolate 346; however, *L. braziliensis* isolate 346 was the only isolate with downregulated GSH1 mRNA levels. This downregulation diminishes the functional relevance of MRPA upregulation. Reduced thiols can also support the Sb susceptibility phenotype observed in isolate 346. Despite the previous link between transporters ABCI4/ABCG2 and Sb resistance,^14^ ^,^ ^15^ we were unable to confirm this phenotype in *L. braziliensis* clinical isolates. In fact, the Sb-sensitive isolate 426 presented downregulation of ABCI4 mRNA levels. The link between ABCG2/ABCI4 and Sb resistance was reported for *L. major*,^14^ ^,^ ^15^ which could be considered a species-specific feature. An observation that supports this hypothesis is the fact that AQP1 was upregulated in *L. major* clinical isolates.^24^

Recent studies headed by Dr Joachim Clos (BNITM, Germany) identified and functionally validated the overexpression of ARM56/ARM58 in SbR *Leishmania* spp.^16^ ^,^ ^25^ This overexpression was first identified by functional cloning using SbR libraries derived from *L. braziliensis* clinical isolates followed by heterologous expression and validation in *L. infantum*.^16^ ^,^ ^25^ ARM56 and ARM58 are part of a subtelomeric cluster on chromosome 34 and can be co-amplified in SbR parasites, favouring drug extrusion mediated by extracellular vesicles.^16^ Here, we identified increased ARM56/ARM58 transcripts in ‘natural’ SbR selected *L. braziliensis*, confirming their previous observation and reinforcing the usefulness of functional cloning based on libraries made from clinical isolates. The mechanistic way ARM56/ARM58 and AQP1 are regulated in *L. braziliensis* has yet to be described; however, because the gene regulation in trypanosomatids is post-transcriptional, the control relies on RNA processing, translation, and degradation.^26^ Drug pressure (or a given environmental selection) could trigger gene amplification or locus deletions that directly leads to increased or reduced/absent mRNA levels, respectively.^10^ These genomic rearrangements are a result of homology guided amplification of gene deletions.^27^ At the transcriptional level, RNA could be controlled by epigenetic markers, leading to increased mRNA steady state or promoting resection.^26^

In addition to molecular marker characterisation in SbR *L. braziliensis*, we also focused on Sb transport studies inspired by the higher intracellular Sb content in Sb-sensitive *L. braziliensis* isolate 346. We wonder if surface glycoconjugates would interfere with Sb uptake. Glycoconjugates play an important role in parasite virulence and infectiveness. The most abundant molecule in *Leishmania* glycocalyx is the LPG, abundant in promastigotes but downregulated in amastigote forms.^28^ ^,^ ^29^ LPG agglutination mediated by CA7AE seems to favour Sb uptake in parasites exposed up to 30 min. After 1 h exposure, the difference is no longer maintained and could be associated with LPG turnover time. The interference of LPG in Sb uptake was validated using a GPI-14 knockout *L. infantum* Ba262 Δ*lpg* strain, which lacks LPG on its surface.^17^ Followed by 1 h treatment, Sb was detected only in the Ba262 mutant. The higher Sb uptake observed for *L. braziliensis* was in accordance with previous observations showing that LPG expression in this species is 10- to 20-fold lower than that in *L. infantum*.^30^ Thus, those preliminary observations provide evidence that surface glycoconjugates may interfere with Sb uptake, and further experiments are being performed.

In summary, simple molecular phenotyping, including drug transport studies and customised transcript level measurements, can be used to identify Sb resistance in *Leishmania* field isolates, helping determine treatment choice and outcome. Predicting Sb resistance in *L. braziliensis* field isolates could direct the chosen first-line treatment away from glucantime. Because drug resistance is a multifactorial phenomenon, different potential molecular markers must be combined and considered for the development of predictive assays applied to disease surveillance and to avoid resistance spreading. From all parameters analysed, we only observed a clear correlation in Sb resistance with higher expression of the ARM56/ARM58 genes. However, other differences could also account for parasite variability. The limited sampling of only two isolates can be unrepresentative and not enough to stablish a pattern of markers for Sb resistance. It could also be species-specific or varies according to geographical regions, depending on where the isolates were obtained from (even classified as same species). Reduced AQP1 mRNA expression in combination with increased ARM58 mRNA and the A516C SNP in the AQP1 gene were sufficient to cluster the SbR clinical isolated *L. braziliensis* mutant. Altogether, these tools could be useful to predict treatment outcome and help control disease and resistance spreading. The next challenge is to apply these techniques to a greater number of isolates from different regions in order to validate these findings and to define the best markers for increasing therapeutic success.
