# Supplementary material for: Antimony resistance in Leishmania (Viannia) braziliensis clinical isolates from atypical lesions associates with increased ARM56/ARM58 transcripts and reduced drug uptake
Source: Mem Inst Oswaldo Cruz. 2019 Aug 15;114:e190111. doi: 10.1590/0074-02760190111 (PMC6697410; doi:10.1590/0074-02760190111)
Supplement: Supplementary file 1 [file 1678-8060-mioc-114-e190111-s.pdf]

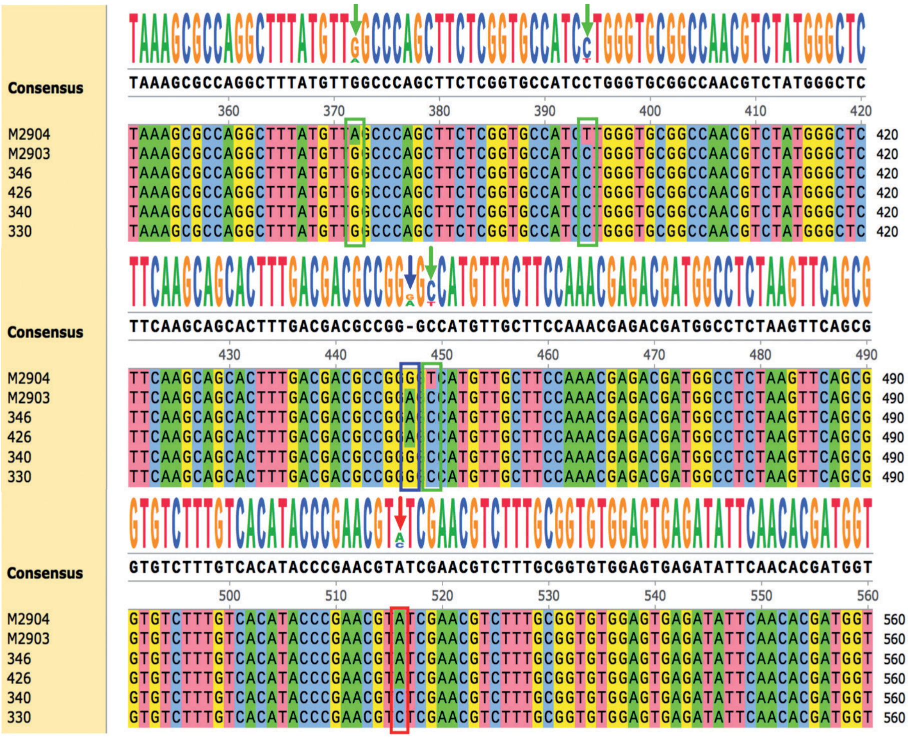

Partial aquaglyceroporin 1 coding gene sequence alignment from *Leishmania braziliensis* field isolates and reference strains. Most single nucleotide polymorphisms (SNPs) were silent mutations except mutation T449C in typical lesion-derived *L. braziliensis* that leads to V150A in protein sequence. Red rectangle: SNP exclusive from AT lesion-derived *L. braziliensis* (A516C); Blue rectangle: SNP present in typical lesion-derived parasites, shared with reference strain M2903; Green rectangles: SNPs present in all field isolates that differs from M2904 reference strain. Complete sequence of AQP1 gene contain 858 nucleotides, here is shown only the polymorphic region ranging from nucleotide positions 351-560.

CC BY
